# The Prevalence and Determinants of Chronic Non-Communicable Disease Risk Factors amongst Adults in the Dikgale Health Demographic and Surveillance System (HDSS) Site, Limpopo Province of South Africa

**DOI:** 10.1371/journal.pone.0147926

**Published:** 2016-02-16

**Authors:** Eric Maimela, Marianne Alberts, Sewela E. P. Modjadji, Solomon S. R. Choma, Sekgothe A. Dikotope, Thembelihle S. Ntuli, Jeane-Pierre Van Geertruyden

**Affiliations:** 1 Department of Medical Sciences, Public Health and Health Promotion, University of Limpopo (Turfloop Campus), Polokwane, South Africa; 2 International Health Unit, University of Antwerp, Antwerp, Belgium; Institute of Infectious Disease and Molecular Medicine, SOUTH AFRICA

## Abstract

**Background:**

The aim of the study was to determine the prevalence and determinants of chronic non-communicable disease (NCD) risk factors in a rural community in the Limpopo Province of South Africa.

**Methods:**

This survey was conducted using the WHO "STEPwise approach to the surveillance of non-communicable diseases" (STEPS) methodology. Participants were residents of the Dikgale HDSS site and standardised international protocols were used to measure behavioural risk factors (smoking, alcohol consumption, fruit and vegetable intake and, physical activity) and physical characteristics (weight, height, waist and hip circumferences and blood pressure–BP). Fasting blood glucose, triglyceride, cholesterol and HDL-C were determined in 732 participants. Data were analysed using STATA 12 for Windows.

**Results:**

The prevalence of current smokers amongst the participants was 13.7%, of which 81.3% were daily smokers. Alcohol was consumed by 16.3% of the participants. The majority of participants (88.6%) had low daily intake of fruit and vegetables and low physical activity (66.5%). The prevalence of hypertension amongst the participants was 38.2%. Overweight, obesity and high waist circumference were prevalent in females. The cardio-metabolic risk profile was not significantly different between men and women. People who were older than 40 years, overweight or obese and those who consumed alcohol were more likely to be hypertensive. Smoking was associated significantly with older age, males, never married and divorced people. Alcohol consumption was associated with older age, males, low educational status and low income.

**Conclusion:**

High levels of risk factors for NCDs among adults in the Dikgale HDSS suggest an urgent need for health interventions to control these risk factors at the population level in order to reduce the prevalence of NCDs.

## Introduction

Non-communicable diseases (NCDs) are amongst the leading causes of death in the world as they result in high mortality rates [1]. Almost all countries are experiencing an increase in the NCDs, which affects all age groups; both poor and rich people and men and women [2]. Currently NCDs represent 43% of the global burden of disease, which indicates an emerging epidemic as deaths as a result of NCDs are predicted to rise to between 60% and 70% of all deaths in 2020 [3, 4]. Evidence shows that the burden of NCDs in South Africa has increased over the past 15 years, resulting in an estimated 37% of all-cause mortality and 16% of disability-adjusted life years [5, 6]. It has been reported that mortality due to NCDs is similar in all Provinces of South Africa, even though the causes may be different [7].

The available data in the scientific world reveal that nearly 80% of NCD-related deaths occur in low- and middle-income countries, despite the swift development of NCDs in high income countries. Most of the impacts resulting from mortality related to NCDs are preventable through innovative interventions which are cost-effective and achievable [1].

Most NCDs share common risk factors, which are often categorised as behavioural or biological [8]. Tobacco use, excessive alcohol consumption, an unhealthy diet and physical inactivity are the behavioural risk factors which contribute to the development of non-communicable diseases [8–10]. Low- and middle-income countries have the highest prevalence of these risk factors and people of low socio-economic status are mostly affected [1].

An insight into the extent of the burden of risk factors for chronic non-communicable diseases in rural communities in the Limpopo Province of South Africa is crucial for effective advocacy and action. This could mainly contribute to the establishment of concerted and inclusive actions to improve chronic disease management irrespective of the cause by closely linking the development and global health agendas [11]. Surveillance of the major modifiable NCD risk factors in the population is indispensable to the planning, implementation and evaluation of health programmes using good policies [12, 13]. Therefore, the aim of this study was to determine the prevalence of risk factors for non-communicable diseases and to identify their demographic and behavioural determinants in the Dikgale Health and Demographic Surveillance System (HDSS) centre.

## Materials and Methods

The Dikgale Health and Demographic Surveillance System (HDSS) centre [14] consists of 15 villages situated close to one another, with a total population of approximately 36 000. For the purpose of determining risk factors for chronic diseases, the population was divided into permanent and migrant subjects. Based on the situation in the old field site, it was estimated that 20% of the population in the 20–59 age group were migrant workers [15]. A person was classified as a migrant if he/she spent less than six months/year in the Dikgale HDSS but still regarded Dikgale as his/her home. As a result, migrants were excluded from taking part in the study because chronic infectious and non-infectious disease risk factors in these two populations are different. Assuming a confidence of 95%, a margin of error of 5% and a conservative prevalence estimate of 50%, the initial sample size was set at 380 per age group. No adjustment for design effect was required as, based on HDSS database, an individual sample frame was used. For each age group the sample size was adapted using a Finite Population Correction (FPC) [16]. A total of 2 981 participants were randomly selected to take part in the STEPwise approach to chronic disease risk factor surveillance from the DHSS database. A total of 1 407 people (878 women and 525 men) completed the WHO STEPwise questionnaire, while only 817 of the participants were available to donate a fasting blood sample as many participants left for work early in the morning. The reasons for not participating included, amongst others, not at home after repeated visits, refusal, death and migrated out of the study area. A total of 85 participants who were HIV positive were excluded from the biochemical data analysis and thus a total of 732 participants formed the study population.

**Fig 1:**
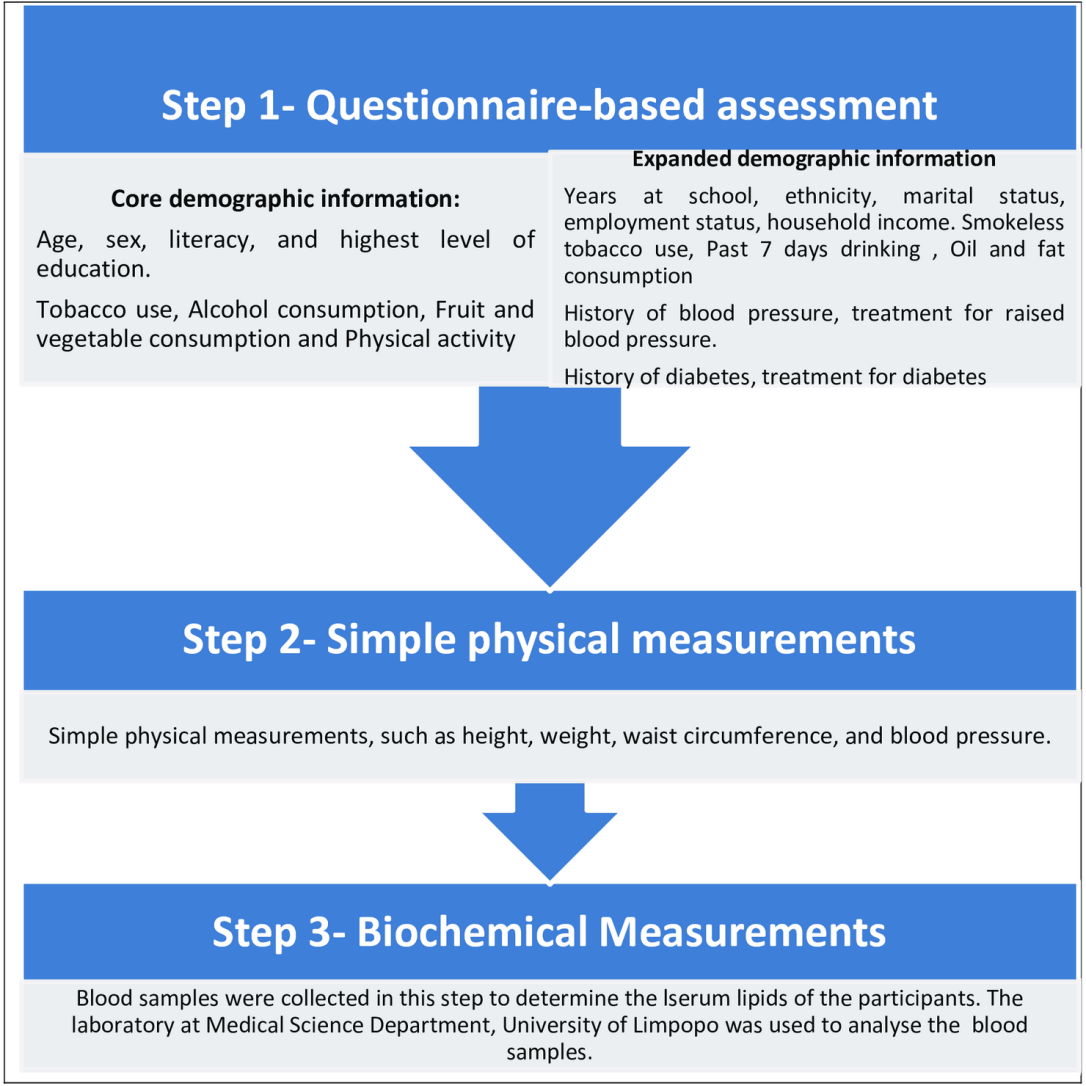
Data collection process using STEP instrument for WHO STEPwise approach to surveillance.

The participant’s demographic information, such as age, gender and educational achievement, were extracted from the Dikgale HDSS database. The WHO STEPwise approach to Surveillance (STEPS) for NCD risk factors [15] was used to collect information on behavioural risk factors through face-to-face interviews; and physical measurements were conducted following the recommended STEPwise protocols [17, 18], as illustrated in Fig 1. The OMRON M6 and M5-I Digital Automatic Blood Pressure Monitors were used to measure resting blood pressure. Blood pressure was measured three times and the average of the last two readings used [19–21].

Criteria used for the diagnosis of hypertension were those proposed by the World Health Organisation (WHO)/International Society of Hypertension, using the average systolic BP of 140mmHg or higher, or if the average diastolic BP was 90mmHg or higher if participants were on anti-hypertensive treatment [20–23]. Height and weight were measured once using a stadiometer and digital balance respectively. The readings were recorded to the nearest 0.5 centimetre and to the nearest 0.1 kg, respectively. Participants were weighed and measured without shoes and wearing only light clothing. Fasting blood glucose and total cholesterol were measured using ILAB 300 and the cut off values used were as follows: High fasting Blood Glucose ≥ 7mmol/l, Total Cholesterol ≥ 5mmol/l, Low HDL Cholesterol <1.00 mmol/l for men and <1.30 mmol/l for women, Triglycerides ≥1.70 mmol/l and TCHOL/HDL-C ratio >5.3. Waist circumference was measured once using a constant tension tape and recorded to the nearest 0.1 cm (High Waist Circumference >102 cm for men and >88 cm for women) [24].

### Statistical Methods

STATA statistical software (STATA Corporation, College Station, Texas) was utilised for all analyses and categorical variables were presented as percentages, whilst continuous variables were expressed as mean ± SD. The coding of data was done in line with WHO guidelines [19]. Comparison of categorical variables was performed using Chi-Square and a level of 0.05 was considered significant. We reported 95% confidence intervals (95% CIs) on all proportions. Univariate logistic regression was used to develop a multivariate logistic regression model to quantify the determinants of behavioural and biomedical risk factors [25].

### Ethical Consideration

Ethical approval of the study was obtained from the Medunsa Research and Ethics Committee (MREC) at University of Limpopo and Department of Health of the Limpopo Province. The Dikgale Tribal Authority gave permission for the study to be conducted and signed full informed consent forms were completed from each participant.

## Results

The mean ages of men and women were 41.29±21.46 years and 45.74±20.39 years respectively. Sixty two percent of the participants were females and most of the participants were never married (55%), while 58% had low education. Literacy amongst men and women was 39.6% and 43.3% respectively, while the employment rate amongst men and women was 32.4% and 26.8% respectively. The definitions of variables used in the results are explained in Table 1. Obesity was more prevalent in females than in males and overall Body Mass Index was significantly different between men and women (Table 2).

**Table 1:** Definition of variables used in results section.

**MET**	Metabolic equivalent (MET) is the ratio of a person's working metabolic rate relative to the resting metabolic rate. One MET is defined as the energy cost of sitting quietly, and is equivalent to a caloric consumption of 1 kcal/kg/hour.
**Moderate intensity physical activity**	Refers to activities which take moderate physical effort and that make a person breathe somewhat harder than normal. Examples include cleaning, vacuuming, polishing, gardening, cycling at a regular pace or horse-riding. Moderate intensity activities require an energy expenditure of approximately 3–6 METs.
**Vigorous intensity activity**	Refers to activities which take hard physical effort and which make a person breathe much harder than normal. Examples include loading furniture, digging, playing football, tennis or fast swimming. Vigorous activities require an energy expenditure of greater than 6 METs.
**Risk Factor**	Refers to any attribute, characteristic, or exposure of an individual, which increases the likelihood of developing a disease, or other unwanted condition/event.
**Serving (of fruit or vegetable)**	For vegetables this refers to one cup of raw, leafy green vegetables, (spinach, salad etc.), one half cup of other vegetables, cooked or raw (tomatoes, pumpkin, beans etc.), or a half cup of vegetable juice. For fruit, this refers to one medium-sized piece of fruit (banana, apple, kiwi etc.) or a half cup of raw, cooked or canned fruit or a half cup of juice from a fruit (not artificially flavoured).
**Alcohol consumption**	Alcohol consumption status of all respondents. Abstainers have not consumed alcohol in the last 12 months.
**Literacy**	Proportion of respondents being able to read and write. Instrument question: Can you read and write?
**Highest level of education**	Highest level of education achieved by the survey respondents. Instrument question: What is the highest level of education you have completed?

**Table 2:** Characteristics of participants Dikgale HDSS, South Africa.

	Females (n = 878)		Males (n = 525)		P-value for trend
	Mean	±SD	Mean	±SD	
Age (years)	45.7	20.3	41.3	21.5	<0.001
Waist (cm)	87.6	14.4	85.5	17.1	0.63
SBP (mmHg)	125.5	24.3	123.8	22.9	0.26
DBP (mmHg)	80.8	12.7	81.9	13.3	0.17
BMI (kg/m2)	**n**	**%**	**n**	**%**	<0.001
Normal 18.5–24.9	309	39.5	308	65.5	<0.001
Overweight 25–29.9	225	28.7	115	24.5	<0.001
Obese ≥30	249	31.8	47	10.4	<0.001
Low education	496	56.7	317	60.4	<0.001
Not working	379	43.3	208	39.6	<0.001
**Marital status**					
Never married	437	50.1	327	62.5	<0.001
Married	327	37.5	172	32.9	<0.001
Divorced	18	2.1	17	3.3	<0.001
Widowed	90	10.3	7	1.3	<0.001

### Prevalence of behavioural risk factors

#### Smoking

The overall prevalence of tobacco smoking (current smokers) was 13.7% and was significantly higher in males 29.2% than in females 4.5% (*p<0*.*001*). Amongst current smokers, 81.3% were daily smokers (90.3% in males compared to 46.2% in females (*p<0*.*001*)) (Table 3). The prevalence of the use of smokeless tobacco products was 10.9% of the total population. More female participants (15.7%) used smokeless tobacco products than males (2.4%) (*p<0*.*001)* (Table 3).

**Table 3:** Prevalence of behavioural risk factors for NCDs by gender.

	Both sexes	Males (n = 528)	Females (n = 876)	P-value for trend at (males vs females)
	% (95% CI)	% (95% CI)	% (95% CI)	
**Smoking**				
Current smokers	13.7 (11.9–15.5)	29.2 (25.3–33.1)	4.5 (3.1–5.8)	<0.001
Daily smokers	81.3 (75.8–86.9)	90.3 (85.5–95.0)	46.2 (30.2–62.1)	<0.001
Smokeless tobacco products	10.9 (9.2–12.6)	2.4 (0.9–3.8)	15.7 (13.2–18.3)	<0.001
Smokeless tobacco daily	92.4 (87.5–97.2)	90.9 (72.9–108.9)	92.5 (87.5–97.6)	0.330
**Alcohol consumption**				
Consume last 12 months	16.3 (14.3–18.2)	28.9 (25.1–32.9)	8.6 (6.8–10.5)	<0.001
Alcohol in 30days	84.4 (78.9–89.9)	88.5 (82.5–94.4)	75.9 (64.3–87.5)	0.036
**Low fruit and vegetables**				
<5 servings/day	88.6 (87.0–90.4)	88.8 (86.1–91.5)	88.6 (86.5–90.7)	0.91
**Physical activity**				
Low (<600 MET-min)	66.5 (63.9–68.9)	40.7 (36.5–44.9)	70.8 (67.8–73.8)	<0.001
Moderate (600–2999 MET-min)	33.5 (31.1–36.0)	59.3 (55.1–63.5)	29.2 (26.2–32.2)	<0.001

The mean age of starting to smoke was 25.3±18.4 years. In the total population, the prevalence of smoking (current smokers) was significantly higher in older males (from 13.0% in age group 15–25 years to 58.3% in age group 45–54 years) (*p<0*.*001)* than in females. The prevalence of the use of smokeless tobacco products was significantly higher amongst older people of both genders (*p<0*.*001)*. In females, use of smokeless tobacco products increased significantly from 1.1% in age group 15–25 years to 26.6% in age group 65 years and above (*p<0*.*001)* (Table 4).

**Table 4:** Prevalence of behavioural risk factors for NCDs by sex stratified by age groups.

Females (n = 878)							
Risk factor	15–24 years % (95% CI)	25–34 years % (95% CI)	35–44 years % (95% CI)	45–54 years % (95% CI)	55–64 years % (95% CI)	≥65 years % (95% CI)	P-value
**Smoking**							
Current smokers	2.5 (0.3–4.7)	4.9 (1.1–8.8)	4.0 (0.1–7.9)	2.8 (0.08–5.6)	5.6 (1.5–9.6)	6.9 (3.3–10.6)	0.06
Daily smokers	2.0 (0.01–3.9)	1.6 (-0.6–3.9)	1.0 (-0.9–2.9)	2.8 (0.1–5.6)	2.4 (-2.9–5.0)	2.1 (0.0–4.2)	0.65
Smokeless tobacco products	1.1 (-0.4–2.7)	4.5 (0.6–8.3)	11.9 (5.3–18.6)	22.6 (15.6–29.7)	26.2 (18.4–34.1)	26.6 (19.9–33.2)	<0.001
**Alcohol consumption**							
Ever consumed alcohol	3.0 (0.6–5.4)	12.2 (6.4–18.0)	6.1 (1.3–10.8)	10.7 (5.6–15.9)	14.9 (8.7–21.2)	13.9 (8.9–18.9)	0.001
Consume last 12 months	3.0 (0.6–5.4)	12.2 (6.4–18.0)	3.0 (-0.3–6.4)	9.2 (4.4–13.9)	12.6 (6.8–18.4)	12.2 (7.5–16.9)	0.001
Alcohol in 30 days	50.0 (-7.9–107.9)	64.3 (37.6–90.9)	100	71.4 (34.4–108–4)	83.3 (65.4–101.2)	87.5 (70.4–104.6)	0.005
**Low fruit and vegetables**							
<5 servings/day	91.5 (87.6–95.4)	86.9 (81.0–92.4)	92.9 (87.8–98.0)	88.0 (82.7–93.4)	85.8 (79.7–91.9)	86.7 (81.8–91.6)	0.08
**Physical activity**							
Low (<600 MET-min)	58.8 (51.9–65.7)	69.9 (61.8–78.1)	74.7 (66.1–83.4)	71.8 (64.4–79.3)	78.7 (71.6–85.9)	75.5 (69.4–81.7)	<0.001
**Males (n = 525)**							
Risk factor	15–24 years % (95% CI)	25–34 years % (95% CI)	35–44 years % (95% CI)	45–54 years % (95% CI)	55–64 years % (95% CI)	≥65 years % (95% CI)	P-value
**Smoking**							
Current smokers	13.0 (8.2–17.9)	25.9 (16.1–35.9)	50.0 (33.4–66.6)	58.3 (44.2–72.5)	41.0 (30.0–52.0)	29.4 (20.5–38.3)	<0.001
Daily smokers	12.5 (7.7–17.3)	23.4 (13.8–32.9)	50.0 (33.4–66.6)	50.0 (35.7–64.3)	37.2 (26.4–47.9)	25.5 (16.9–34.0)	<0.001
Smokeless tobacco products				2.5 (-0.4 (7.4)	4.3 (-0.5–9.1)	7.3 (2.0–12.5)	<0.001
**Alcohol consumption**							
Ever consumed alcohol	16.9 (11.5–22.4)	36.4 (25.5–47.2)	41.7 (25.3–58.0)	52.1 (37.8–66.4)	44.9 (33.7–56.0)	41.2 ((31.6–50.8)	<0.001
Consume last 12 months	15.8 (10.5–21.1)	32.5 (21.9–43.0)	33.3 (17.7–48.9)	43.8) 29.5–57.9)	37.2 (26.4–47.9)	34.3 (25.0–43.6)	<0.001
Alcohol in 30 days	83.3 (65.4–101.2)	80.0 (61.8–98.2)	100	91.7 (75.2–108.2)	88.0 (74.9–101.1)	93.5 (84.7–102.4)	0.101
**Low fruit and vegetables**							
<5 servings/day	93.5 (89.9–97.1)	93.5 (87.9–99.1)	88.9 (788.5–99.3)	87.5 (78.0–96.9)	85.9 (78.1–93.7)	80.4 (72.6–88.2)	0.001
**Physical activity**							
Low (<600 MET-min)	51.6 (44.4–58.9)	59.7 (48.7–70.8)	52.8 (36.2–69.3)	52.1 (37.8–66.4)	62.8 (52.0–73.6)	75.5 (67.1–83.9)	<0.001

#### Alcohol consumption

The prevalence of alcohol consumption (consumed alcoholic drinks in last 12 months) was 16.3% of the total population and males had a significantly higher prevalence for alcohol consumption than females 28.9% vs 8.6% (*p<0*.*001*) respectively (Table 3). The prevalence of alcohol consumption (consumed alcoholic drinks in last 12 months) in males increased from 16.9% in age group 15–25 years to 44.9% in age group 55–64 years (*p<0*.*001*) but a decline of 3.7% in age group 65 years and above was noticed (*p<0*.*001*) (Table 4).

#### Low fruit and vegetable intake

The prevalence of low fruit and vegetable intake (less than 5 servings of fruits and vegetables, which is equivalent to at least 400g of fruit and vegetables per day) was 88.6% for both sexes and there was no significant difference between males and females (*p = 0*.91) (Table 3). The prevalence of low fruit and vegetable intake was significantly lower in older males (80.4%) in age group 65 years and above, compared to 93.3% in the age group 15–24 years (*p<0*.*001)*. Among females there was no trend in the prevalence of low fruit and vegetable intake with age, *p = 0*.*08* (Table 4).

#### Low physical activity

The prevalence of low physical activity, which is (MET-minute/week <600MET in a form of work, travel to and from places, recreational activities), was found in 65.5% of the total population. Females had a significantly higher prevalence for physical inactivity 70.8% as compared to 40.7% for males, *p<0*.*001* (Table 3). The prevalence of low physical activity increased significantly with age in females, from 58.8% in age group 15–25 years to 78.7% in age group 55–64 years, *p<0*.*001*. A similar trend was seen in males (Table 4).

### Prevalence of hypertension, anthropometric and biochemical risk factors

#### Hypertension

The overall prevalence of hypertension was found to be 38.9% of the total population and there was no significant difference between males and females, *p = 0*.*27*. The prevalence of hypertension increased significantly with age in females (from 29.4% in age group 15–25 years to 49.5% in age group 65 years and above, *p<0*.*001*). A similar trend was observed in males (from 32.6% in the 15–25 year age group to 49.5% in age group 65 years and above, *p = 0*.*003)* (Table 5).

**Table 5:** Prevalence of physical risk factors for NCD by gender stratified by age groups.

**Females**								
Risk factor	15–24 years % (95% CI)	25–34 years % (95% CI)	35–44 years % (95% CI)		45–54 years % (95% CI)	55–64 years % (95% CI)	≥65 years % (95% CI)	P-value
**Hypertension** (BP>140/90mmHg)	29.4 (23.1–35.8)	31.1 (22.9–39.4)	27.6 (18.6–36.5)		42.9 (34.6–51.1)	45.6 (36.8–54.4)	49.5 (42.3–56.6)	<0.001
**Overweight** (BMI kgm^2^ ≥25 to ≤29.9)	28.8 (22.1–35.5)	28.2 (19.7–36.6)	25.3 (15.9–34.7)		32.1 (24.1–40.0)	23.2 (15.3–31.1)	31.9 (24.8–39.1)	<0.001
**Obesity** (BMI kgm^2^ ≥30)	13.6 (8.5–18.6)	28.2 (19.7–36.6)	37.3 (26.9–47.8)		40.3 (31.9–48.6)	41.9 (32.8–51.2)	37.3 (29.9–44.7)	<0.001
**High waist circumference** (≥102 men and ≥88 women)	55.7 (48.3–63.2)	56.2 (46.6–65.7)	60.0 (49.5–70.5)		60.2 (51.6–68.7)	58.4 ((49.3–67.5)	52.4 (44.8–60.1)	0.74
**Males**								
Risk factor	15–24 years % (95% CI)	25–34 years % (95% CI)	35–44 years % (95% CI)	45–54 years % (95% CI)		55–64 years % (95% CI)	≥65 years % (95% CI)	P-value
**Hypertension** (BP>140/90mmHg)	32.6 (25.7–39.5)	28.8 (18.3–39.2)	36.1 (20.2–52.1)	35.4 (21.7–49.1)		47.4 (36.0–58.7)	49.5 (39.7–59.3)	0.003
**Overweight** (BMI kgm^2^ ≥25 to ≤29.9)	20.4 (14.2–26.5))	17.5 (7.9–26.9)	28.1 (12.3–43.9)	26.2 (12.7–39.7)		28.6 (17.9–39.3)	32.3 (22.7–41.8)	0.51
**Obesity** (BMI kgm^2^ ≥30)	10.2 (5.6–14.8)	17.5 (7.9–26.9)	3.1 (-3.0–9.3)	9.5 (0.5–18.5)		11.4 (3.9–18.9)	6.5 (1.4–11.5)	0.51
**High waist circumference** (≥102 men and ≥88 women)	31.3 (24.2–38.4)	29.2 (18.1–40.4)	28.1 (12.3–43.9)	38.6 (24.0–53.2)		30.9 (19.8–41.9)	30.1 (20.7–39.5)	0.76

#### Overweight and Obesity

The overall prevalence of overweight was 27.1% of the total population and a high prevalence of overweight was observed in older females (*p<0*.*001)* (Table 5). The prevalence of obesity in females was higher (27.8%) than in males (10.6%) (*p<0*.*001*). In females, obesity was highest in the age group 45–64 years. The prevalence of obesity in females showed an increasing trend from 13.6% in age group 15–25 years to 41.9% in age group 55–64 years. (*p<0*.*001)* (Table 5).

#### High waist circumference

The prevalence of high waist circumference was found to be 34.6% of the total population. Females had a significantly higher prevalence (49.8%) than did males (7.8%) *p<0*.*001*. There was no significant trend in the prevalence of high waist circumference with age (Table 5).

#### High fasting blood glucose

The prevalence of high fasting blood glucose, which was equal to or above 7.0 mmol/L, in the study participants was 12.5% of total population. The prevalence of high fasting blood glucose was significantly higher in older participants and increased from 2.8% in age group 15–25 years to 18.8% in age group ≥65 years in females *p = 0*.*017* and from 1.5% in age group 15–25 years to 19.6% in age group ≥63 years in males *p = 0*.*003* (Table 6). If using a glucose level equal or above 11.1mmol/L or being on treatment for control of blood glucose, the prevalence of diabetes among the study participants was 4%.

**Table 6:** Prevalence of biochemical risk factors for NCD by sex stratified by age groups.

**Females**							
Risk factor	15–24 years % (95% CI)	25–34 years % (95% CI)	35–44 years % (95% CI)	45–54 years % (95% CI)	55–64 years % (95% CI)	≥65 years % (95% CI)	P-value
**High Fasting Blood Glucose** (≥7.0 mmol/l)	2.8 (-11.1–6.7)	1.9 (-1.9–5.7)	6.8 (-0.7–14.4)	9.1 (3.0–15.1)	15.7 (7.1–24.3)	18.8 (10.9–26.6)	0.017
**Raised Triglycerides Levels** (≥1.7 mmol/l)	7.0 (1.0–13.1)	13.7 (4.2–23.3)	18.6 (6.8–30.4)	27.1 (17.5–36.6)	25.7 (15.4–36.1)	39.6–29.7–49.4)	<0.001
**High Cholesterol Levels** (≥5.0 mmol/l)	20.0 (10.5–29.5)	17.3 (6.9–27.7)	22.7 (10.2–35.3)	32.6 (22.6–42.5)	47.1 (35.3–58.9)	48.4 (38.3–58.6)	<0.001
**TC/HDL-C Ratio** (>5.0)	4.3 (-0.5–9.1)	7.8 (0.3–15.3)	6.9 (-0.8–14.7)	12.9 (5.7–20.1)	17.1 (8.2–26.1)	22.6 (14.0–31.1)	<0.001
**Males**							
Risk factor	15–24 years % (95% CI)	25–34 years % (95% CI)	35–44 years % (95% CI)	45–54 years % (95% CI)	55–64 years % (95% CI)	≥65 years % (95% CI)	P-value
**High Fasting Blood Glucose** (≥7.0 mmol/l)	1.5 (-1.5–5.6)	6.9 (-2.5–16.3)		4.3 (-4.2–12.9)	12.2 (2.0–22.4)	19.6 (9.1–30.2)	0.003
**Raised Triglycerides Levels** (≥1.7 mmol/l)	11.1 (3.2–18.9)	37.9 (19.9–56.0)	21.4 (-0.9–43.9)	21.7 (4.4–39.1)	29.3 (15.1–43.4)	40.77 (27.4–54.0)	0.004
**High Cholesterol Levels** (≥5.0 mmol/l)	15.6 (6.6–24.6)	44.4 (25.2–63.6)	7.1 (-6.9–21.2)	21.7 (4.4–39.1)	14.6 (3.6–25.6)	48.2 (34.9–61.5)	<0.001
**TC/HDL-C Ratio** (>5.0)		14.8 (1.1–28.5)	15.4 (-5.1–35.9)	4.5 (-4.4–13.5)	9.7 (0.5–19.0)	28.6 (16.6–40.6)	<0.001

#### Raised triglycerides levels

The overall prevalence of raised triglycerides levels was 25.4%. The prevalence of raised triglycerides levels was significantly higher in older participants of both sexes. Amongst females, the prevalence was 7.0% in age group 15–25 years and 39.6% in age group 65 years and above *(p<0*.*001)*. A similarly increasing trend was observed in males (11.1% in age group 15–25 years to 40.8% in age group 65 years and above) *(p = 0*.*004)* (Table 6).

#### Total Cholesterol and HDL Cholesterol levels

The prevalence of high total cholesterol (TC) levels was 32.6% of total population. Older females showed a higher prevalence of high total cholesterol (TC) levels when compared to younger female participants (20.0% in age group 15–25 years to 48.4% in age group 65 years and above, *p<0*.*001)*. A similarly increasing trend was observed in males from 15.6% in age group 15–25 years to 48.2% in age group 65 years and above, *p<0*.*001* (Table 6). But our results also showed a high prevalence of high total cholesterol (TC) levels in males aged 25–34 years. The overall prevalence of high Total Cholesterol/HDL-cholesterol ratio was 10.6%, with no significant difference between males and females, *p = 0*.*28* (Table 6). There was a higher prevalence of high Total Cholesterol/HDL-cholesterol ratio in older males and females, 4.3% in 15–25 age group to 22.6% in age group 65 years and above in females and from 14.8% in age group 25 – 34years to 28.6% in age group 65 years and above in males respectively, *p<0*.*001* (Table 6).

### Determinants of behavioural risk factors

#### Tobacco use, alcohol consumption, low fruit and vegetable intake and low physical activity

Older people were 3.3 times more likely to be smokers (*p<0*.*001)*, 5.3 times more likely to use smokeless tobacco products (*p<0*.*001)*, 2.4 times more likely to consume alcohol (*p<0*.*005)* and 1.1 times more likely to have low fruit and vegetable intake (*p<0*.*005)* (Table 7). Males were 10.6 times more likely to be smokers (*p<0*.*001)*, 0.1 less likely to use smokeless tobacco products (*p<0*.*001)*, 4.9 times more likely to consume alcohol (*p<0*.*001)* and 0.6 times less likely to be physically inactive (*p<0*.*001)* (Table 7) than were females. People with low education were found to be 2 times more likely to use smokeless tobacco products (*p<0*.*05)* and 0.6 times less likely to consume alcohol (*p<0*.*05)* than were educated people. People who were never married were 2.5 times more likely to be smokers (*p<0*.*001)* than were married people, while divorced people were 5.8 times more likely to be smokers than were married people (*p<0*.*001)* and widowed people were 1.9 times more likely to be smokers. Marital status was not significantly associated with the use of smokeless tobacco products, alcohol consumption or intake of low fruit and vegetables. People in the low income category were 1.4 times more likely to consume alcohol than people in the high income category *(p = 0<0*.*05)* (Table 7).

**Table 7:** Multivariate logistic regression to determine predictors of behavioural risk factors for NCD.

Variables	Smoking	Smokeless tobacco products	Alcohol consumption	Low fruit and vegetable intake	Physical inactivity
	Hosmer-Lemeshow = 0.41 Sensitivity = 24.34% Specificity = 97.75%	Hosmer-Lemeshow = 0.34 Sensitivity = 0.0% Specificity = 100%	Hosmer-Lemeshow = 0.45 Sensitivity = 0.0% Specificity = 100%	Hosmer-Lemeshow = —Sensitivity = 0.% Specificity = 100%	Hosmer-Lemeshow = 0.24 Sensitivity = 0% Specificity = 100%
Age					
15–39 years	Reference (1)	Reference (1)	Reference (1)	Reference (1)	Reference (1)
≥40 years	3.33 (1.99–5.55)***	5.31 (2.45–11.49)***	2.35 (1.39–3.98)**	1.14 (0.69–1.87)	0.87 (0.63–1.22)
Gender					
Female	Reference (1)	Reference (1)	Reference (1)	Reference (1)	Reference (1)
Male	10.63 (7.00–16.13)***	0.14 (0.07–0.27)***	4.89 (3.38–7.07)***	*a*	0.55 (1.22–1.97)***
Educational status					
High	Reference (1)	Reference (1)	Reference (1)	Reference (1)	Reference (1)
Low	0.91 (0.61–1.36)	2.14 (1.25–3.69)*	0.56 (0.36–0.87)*	0.86 (0.57–1.29)	1.08 (0.83–1.42)
Marital status					
Married	Reference (1)	Reference (1)	Reference (1)	Reference (1)	Reference (1)
Never married	2.52 (1.54–4.12)***	0.66 (0.39–1.05)	1.56 (0.97–2.49)	0.65 (0.41–1.03)	1.52 (1.11–2.10)*
Divorced	5.78 (2.43–13.76)***	2.14 (0.87–5.28)	2.15 (0.92–5.03)	1.21 (0.49–3.03)	1.06 (0.49–2.28)
Widowed	1.97 (0.87–4.48)	1.08 (0.62–1.90)	0.66 (0.29–1.49)	1.07 (0.58–1.97)	0.74 (0.42–1.29)
Work status					
Working	Reference (1)	Reference (1)	Reference (1)	Reference (1)	Reference (1)
Not working	1.35 (0.91–2.01)	1.47 (1.02–2.14)*	1.17 (0.78–1.76)	*a*	0.98 (0.74–1.31)
Income					
High income	Reference (1)	Reference (1)	Reference (1)	Reference (1)	Reference (1)
Low income	*a*	*a*	1.36 (1.08–1.71)*	*a*	*a*

Values are reported as odds ratios (95%CI)

* significant *at p<0*.*05*

** significant at p<0.005

*** significant *at p<0*.*001*

*a =* Not significant in univariate model then dropped

### Determinants of biomedical risk factors

#### Hypertension, High fasting blood glucose, High Cholesterol, Raised triglycerides

Older people were 4.7 times more likely to be hypertensive (*p<0*.*001)*, 1.8 times more likely to have high fasting blood glucose (*p<0*.*05)*, 1.7 times more likely to have high cholesterol levels (*p<0*.*005)*, 2.1 times more likely to have a high total cholesterol HDL-C ratio (*p<0*.*05)* and 2.2 times more likely to have raised triglycerides (*p<0*.*001)* than were young people (Table 8). People who were overweight or obese were found to be 1.7 times more likely to be hypertensive than people of normal weight. Smokers were found to be 1.4 times more likely to be hypertensive (*p = 0*.*189)*, 0.5 times less likely to have high cholesterol levels (*p<0*.*05)* and 0.3 times less likely to have high total cholesterol HDL-C ratio (*p<0*.*05)* than were non-smokers. People who consumed alcohol were 1.6 times more likely to be hypertensive (*p<0*.*05)*, and 0.6 times less likely to have raised triglyceride (*p<0*.*05)* than people who did not consume alcohol. People who had low fruit and vegetable intake were found to be 1.8 times more likely to have high fasting blood glucose (*p<0*.*05)* than did people with normal fruit and vegetable intake (Table 8).

**Table 8:** Multivariate logistic regression to determine predictors of biomedical risk factors.

		Models			
Variables	Hypertension	High fasting blood glucose	High cholesterol levels	TC/HDL- Cholesterol ratio	Raised triglyceride
	Hosmer-Lemeshow = 0.04 Sensitivity = 62.54% Specificity = 65.85%	Hosmer-Lemeshow = 0.07 Sensitivity = 0.0% Specificity = 100%	Hosmer-Lemeshow = —Sensitivity = 0.0% Specificity = 100%	Hosmer-Lemeshow = 0.36 Sensitivity = 0.0% Specificity = 100%	Hosmer-Lemeshow = 0.63 Sensitivity = 73.26% Specificity = 53.22%
Age					
15–39 years	Reference (1)	Reference (1)	Reference (1)	Reference (1)	Reference (1)
≥40 years	4.7 (3.2–6.9)***	1.8 (1.1–3.0)*	1.7 (1.2–2.4)**	2.1 (1.2–3.6)*	2.2 (1.5–3.2)***
Overweight/Obesity					
No	Reference (1)	Reference (1)	Reference (1)	Reference (1)	Reference (1)
Yes	1.7 (1.2–2.3)**	*a*	*a*	*a*	*a*
Smoking					
No	Reference (1)	Reference (1)	Reference (1)	Reference (1)	Reference (1)
Yes	1.4 (0.8–2.4)	*a*	0.5 (0.3–0.8)*	0.3 (0.1–0.5)*	*a*
Alcohol consumption					
No	Reference (1)	Reference (1)	Reference (1)	Reference (1)	Reference (1)
Yes	1.6 (1.1–2.5)**	*a*	0.8 (0.5–1.2)	0.5 (0.2–1.2)	0.6 (0.4–0.9)*
Low fruit and vegetable intake					
No	Reference (1)	Reference (1)	Reference (1)	Reference (1)	Reference (1)
Yes	*a*	1.8 (1.0–3.2)*	*a*	*a*	*a*

Values are reported as odds ratios (95%CI)

* significant *at p<0*.*05*

** significant at p<0.005

*** significant *at p<0*.*001*

Hypertension = BP >140/90 mmHg, High fasting blood glucose = ≥7.0 mmol/l, High cholesterol levels = ≥5.0 mmol/l, TC/HDL- Cholesterol ratio = >5.0, Raised triglyceride = ≥1.7 mmol/l, High waist circumference = ≥ 102 men and ≥ 88 women, Overweight/Obesity = BMI kg.m^2^ ≥25 to ≤29.9/ BMI kg.m^2^ ≥30

*a =* Not significant in univariate model then dropped

## Discussion

Our study revealed that an epidemiological transition is occurring in this rural area of the Limpopo Province of South Africa. The present study reported the prevalence of cigarette/pipe smoking at 13% overall, which is similar to another rural area in South Africa (14%) [26], but lower than the reported national prevalence in 1996 of 31% [27]. The reason for this difference when compared to the national prevalence may be as a result of the fact that the SADHS included urban areas where smoking is known to be more common [27].

Our study demonstrated that risk factors for chronic non-communicable diseases are major public health problems, with a high percentage of men using tobacco and alcohol. The study findings reflect the cultural practices where smoking and alcohol consumption are more prevalent in men than in women. The prevalence of smoking and alcohol consumption in our study were found to be 13.7% and 16.3% of the population respectively, which is lower than the prevalence reported in a study comprising South African adults in all nine provinces aged 18 years and older (38% and 21%) [27], and were also lower than the prevalence found in a study conducted by Alberts et al. in the same study area (57.2% and 35.4%) [4]. The prevalence of smoking was almost similar to that found by another finding from a study undertaken in a rural area in the Mpumalanga Province in 2007 [26]. It is important to note that the Alberts et al. study used participants aged above 30 years and, if we had considered participants aged above 30 years in our study, we would have obtained a similar alcohol prevalence of 16%. It is a well-known phenomenon that prevalence of smoking increases with age. In a study conducted in Vietnam by Pham et al., the prevalence of smoking was found to be higher in men than in women, which concurs with the findings of our study [28]. The high prevalence of smoking amongst men also supports the findings of several surveys undertaken elsewhere [29] and raises concerns about environmental tobacco exposure and the influence of paternal smoking on the youth.

The present study showed that smoking was associated with increasing age, male gender, people who were never married, those who were divorced and those who were widowed. This concurs with other studies conducted in rural HDSSs in Asia [30, 31]. The median age at initiation of smoking was 19 years, which is consistent with a study conducted by Sugathan et al. in Kerala, India on the “Behavioural risk factors for non-communicable disease among adults” [32]. In our study, the risk factors for smokeless tobacco product-use were found to be associated with increasing with age, males, low education, widowers, joblessness and low income. This is consistent with a study conducted by Kakde et al. (2012) in a South Asian population [33]. The use of smokeless tobacco products in rural communities is high mainly due to social acceptance and belief regarding its palliative role in treating minor ailments, such toothache [33].

Our study findings also showed a strong association between alcohol consumption and increasing age, gender and low education, which concurs with the findings of other studies [34, 35]. A high consumption of fruit and vegetables reduces the risk of heart disease, BP and some forms of tumour [36]. The prevalence of inadequate fruit and vegetables consumption, defined as less than five servings a day, was high, as was found in other studies [36, 37]. This was found to be the case in a study undertaken in South Africa in 2009, which showed that 80% of adults aged ≥15 year eat fewer fruit and vegetables than recommended [38]. We saw no gender difference with respect to fruit and vegetable consumption, which is characteristic of rural areas with low income [39]. An Italian study reported a low percentage of adolescents eating fruit and vegetables daily [39]. Our study findings show that gender was not associated with low intake of fruit and vegetables, while studies conducted in other INDEPTH HDSS sites did, indeed, show a gender difference [36].

Engaging in physical activity is widely recognised as the primary means of prevention of chronic diseases, as well as for the treatment and rehabilitation of patients. Moreover, physical activity has beneficial effects on an individual’s health and well-being [40]. Our study shows that females are more physically inactive than are men, which concur with studies conducted in nine rural HDSSs in five Asian countries, namely, are Bangladesh, India, Vietnam, Indonesia and Thailand [41, 42]. These findings also concur with findings from the World Health Survey 2003, which found that women were more physically inactive than are men in South Africa [43]. A study conducted in 51 countries (most of which were developing countries), including South Africa, found that, overall, about 15% of men and 20% of women were at risk for chronic diseases due to physical inactivity [43]. The percentage of physically inactive adults in the world is high [40], despite evidence which clearly indicates that physical activity is critical to improve health and quality of life [44]. Physical inactivity is recognised as an independent coronary artery disease risk factor and is recognised as one of six major cardiovascular risk factors. Being physically active significantly reduces the risk of obesity [45].

There may be under-reporting of physically active behaviour in rural communities when using self-report methods [44, 46]. Therefore, the use of objective measures of physical activity, in addition to or in place of, subjective or self-report measures of physical activity, should be promoted in physical activity epidemiology research [46]. A study by Cook et al. reported that rural African women accumulate in excess of 10 000 steps per day as a result of greater involvement in subsistence or lifestyle activities, such as housework, yard work and walking for transport [46].

Our study showed a hypertension prevalence of 38.0% of the population, which is higher than the findings of the 2005 Alberts et al. study, where approximately 25% of participants had hypertension [4]. Our study findings are contrary to the findings of a study conducted in Vietnam by Pham [28], which reported that more men than women were hypertensive. Although the overall prevalence of hypertension showed no significant difference between males and females, the differences were noted between males and females as age increased, which concurs with findings of a study conducted by Anand et al. (2007) in Faridabad, where the prevalence of hypertension in both genders increased with increasing age [40].

Contrary to the findings of a study conducted in Kerala, India [47], our study findings showed no gender difference in the prevalence of hypertension. Other studies of rural communities in Asia also report high and increasing rates of hypertension [48–50]. Obesity is a growing health problem globally and the WHO emphasises the importance of monitoring the prevalence of overweight and obesity in different populations. We observed a high prevalence of overweight and obesity among females, which is consistent with a study undertaken in India [51]. Furthermore, obesity and overweight was found to be high in our study in the age groups 15–34 years, which has been found elsewhere in SA [52]. Similar trends were observed in northern India [47] and in a rural area near NCT Delhi [48].

Obesity was found to be associated with hypertension in our study, which is consistent with findings observed elsewhere in South Africa and in India [47,53]. A high BMI is a risk factor for diabetes and it has been shown that variations in BMI at the population level predict changes in diabetes prevalence [54]. Women had higher odds of being overweight and obese than did men, which is similar to the findings of another study [54]. Nevertheless, the high prevalence of overweight and obesity amongst the people in this rural population is disturbing and is supported by Zhou et al., 2012, who found that, in South Africa, overweight and obesity are prevalent amongst almost all population and age groups, especially in the rural areas [55].

The prevalence of high fasting blood glucose generally increased with age in all groups assessed in this study, indicating diabetes, at a level of more than 7 mmol/l, in 12.5% of population. This is consistent with a study conducted by Bradshaw et al. in South Africa [9] and a study conducted on a U.S population by Cowie et al. [56], in which the prevalence of high fasting blood glucose generally increased with age in all groups. This high fasting blood glucose amongst the study participants might not be a true reflection of the prevalence of diabetes because fasting might not have been adhered to, as required, and it was difficult to measure adherence. High fasting blood glucose was associated with older age, low education, and the unemployed in our study.

Males had a lower prevalence of high cholesterol levels than did females in our study, which is different from the results of a study conducted by Al-Nuaim in Saudi Arabia [57]. The prevalence of high cholesterol in our female study participants increased with age, reaching a peak of 43.2% in age group 65 years and above, which is consistent with study by Al-Nuaim [57]. In our study, high cholesterol levels were significantly associated with older age, low education; people who were never married and unemployed people. High total cholesterol and low high-density lipoprotein (HDL) cholesterol are major risk factors for coronary heart disease, including heart attacks [58]. Raised serum cholesterol is a modifiable risk factor which is linked to an estimated 4.4 million deaths each year, globally. Raised serum cholesterol is responsible for a sizable number of ischemic strokes and heart disease in all countries [58]. Interventions, such as therapeutic lifestyle changes, are essential in proposals to lower cholesterol, which include, amongst others, minimising dietary intake of saturated fats, controlling body weight and becoming more physically active [58].

In the present study, the prevalence of total-to-HDL cholesterol ratio, a predictor of cardiovascular disease, showed no significant difference between males and females, which concurs with a study conducted in the same area in 2005 by Alberts et al. [4]. In both our study and the study by Alberts et al., there was significant increase in the prevalence of total-to-HDL cholesterol ratio with increasing age in both genders. Hypertriglyceridemia was prevalent in approximately 24.8% of the sample population, while the findings from studies conducted in Angola (10.6%) and Nigeria (15.0%) a lower prevalence of hypertriglyceridemia. The prevalence of hypertriglyceridemia found in the third National Survey on Health and Nutrition (NHANES III), involving African-American men and women, was 21.0% and 14.0%, respectively [59]. In our study, the prevalence of hypertriglyceridemia was similar between men and women. More women had a high waist circumference than men, which is consistent with other studies [59,60].

### Limitations of the study

#### Representativeness of our results

Our study findings need to be interpreted cautiously as the Dikgale HDSS is developed as a sub district level surveillance system in a rural setting in the Capricorn District of the Limpopo Province of South Africa. The survey was cross-sectional and was not conducted throughout the year. Some variables were self-reported, which may have resulted in self-report bias and we did not compute measures of agreement between self-reported conditions and those obtained from actual measurements. Again, some of the behaviours that vary seasonally (e.g., dietary intake) may not be representative; and cause and effect cannot be determined for associations between BMI and selected health conditions. Participants were requested not to eat anything after a certain time the previous evening before they came to participate in the study the following morning but it was difficult to measure adherence to this request, which is a limitation and a challenge to validity on the serum lipids and glucose results.

It was not possible, therefore, to extrapolate our findings to a larger population at provincial or country level. However, the findings in our study are in line with most of the findings from other sub-national or national surveys from other parts of the world. Based on previous surveys, we expected a non-response rate of 10% but our study had a high proportion of non-respondents (32.6%), most of them were from the age group 25–54 years, which represents the working age group, contributing 55.4% of the non-respondents. Using the approach by firstly excluding the people who moved out of the community, deceased, therefore, the main reasons for the non-respondents were absent from home even after repeated visits (27.2%), refused to participate (35.1%), sick (1.3%), underage (2.1%) and prison (0.3%).

## Conclusion

The present study found that the prevalence of risk factors for non-communicable diseases, such as smoking, alcohol consumption, low fruit and vegetable consumption, physical inactivity, hypertension, overweight and high waist circumference is high. Due to the epidemiological transition which is occurring in this rural area, it is recommended that health interventions which aim to control risk factors at population level should be planned and implemented by the provincial government in order to slow the progress of the coming non-communicable diseases epidemic. In conclusion, our findings highlighted the importance of reaching out to poor rural communities with messages regarding the impact of diet, smoking and alcohol consumption on general health.

### Implications of the study

This study in the Dikgale HDSS provides baseline data on risk factors for NCDs and these epidemiological data will be of value if used by the health policy makers in developing interventions for chronic disease risk factor prevention and control [54, 55] in the Limpopo Province. This study suggests that chronic NCDs are common amongst adults in rural areas and, therefore, we propose that primary health care services increasingly accommodate screening and treatment for chronic NCDs and NCD risk factors.
